# Bacterial succession along a sediment porewater gradient at Lake Neusiedl in Austria

**DOI:** 10.1038/s41597-019-0172-9

**Published:** 2019-08-30

**Authors:** Avril Jean Elisabeth von Hoyningen-Huene, Dominik Schneider, Dario Fussmann, Andreas Reimer, Gernot Arp, Rolf Daniel

**Affiliations:** 1 Genomic and Applied Microbiology and Göttingen Genomics Laboratory, Institute of Microbiology and Genetics, Georg-August-University Göttingen, Göttingen, Germany; 2 Geobiology, Faculty of Geosciences and Geography, Georg-August-University Göttingen, Göttingen, Germany

**Keywords:** Sequencing, Microbial ecology, Environmental microbiology

## Abstract

We provide bacterial 16S rRNA community and hydrochemical data from water and sediments of Lake Neusiedl, Austria. The sediments were retrieved at 5 cm intervals from 30–40 cm push cores. The lake water community was recovered by filtration through a 3.0/0.2 µm filter sandwich. For 16S rRNA gene amplicon-based community profiling, DNA was extracted from the sediment and filters and the bacterial V3-V4 regions were amplified and sequenced using a MiSeq instrument (Illumina). The reads were quality-filtered and processed using open source bioinformatic tools, such as PEAR, cutadapt and VSEARCH. The taxonomy was assigned against the SILVA SSU NR 132 database. The bacterial community structure was visualised in relation to water and porewater chemistry data. The bacterial community in the water column is distinct from the sediment. The most abundant phyla in the sediment shift from *Proteobacteria* to *Chloroflexota* (formerly *Chloroflexi*). Ammonium and total alkalinity increase while sulphate concentrations in the porewater decrease. The provided data are of interest for studies targeting biogeochemical cycling in lake sediments.

## Abstract (summary)

**Table:** 

Design Type(s)	source-based data analysis objective • biodiversity assessment objective
Measurement Type(s)	freshwater metagenome
Technology Type(s)	DNA sequencing
Factor Type(s)	Environment • depth
Sample Characteristic(s)	metagenome • Neusiedlersee • lake

Machine-accessible metadata file describing the reported data (ISA-Tab format)

## Background & Summary

Lake Neusiedl is the largest, seasonally evaporative lake in western Europe covering an area of approximately 315 km^2^ ^1^. Its sediments show high contents of authigenic high magnesium calcite and poorly ordered dolomite, which have been the focus of multiple studies on sediment formation, geochemistry and water level^1–4^. There is a strong economic interest in the lake and the surrounding national parks due to their recreational value^1,5^. Thus, the lake’s water quality, including potential pathogenic microbes, is monitored on a regular basis^6–9^. Nevertheless, the bacterial community composition of water and sediment remains largely unexplored, particularly in relation to the lakes’ hydrochemistry.

**Fig. 1:**
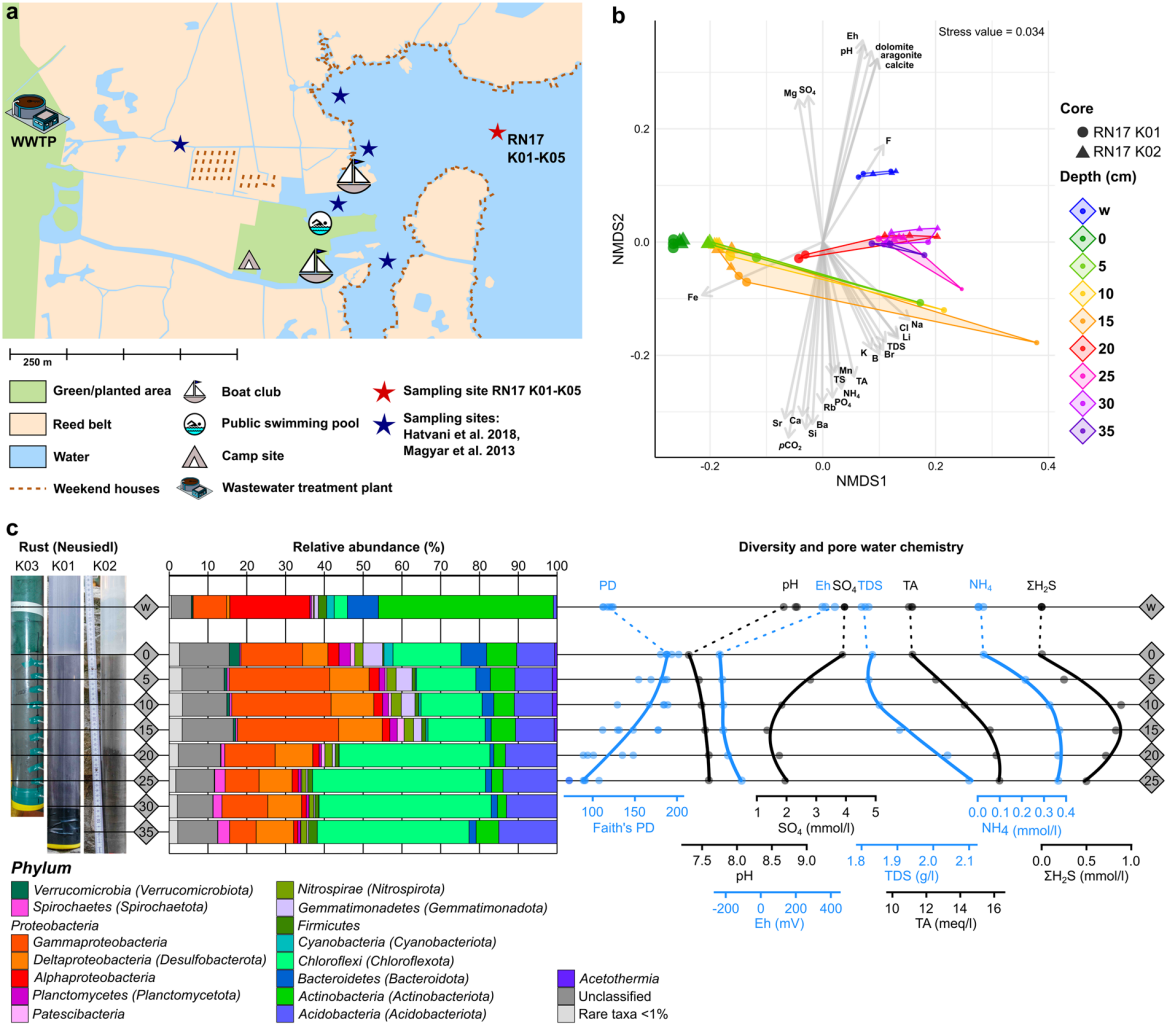
Sampling site in the bay of Rust, NMDS and depth profiles of the bacterial community composition and porewater properties. (**a**) Sampling site of this study (red star) and previous studies (blue stars). Markers for anthropogenic influences, such as a wastewater treatment, holiday houses (brown dashed lines) and recreational sites (pool, boat club, camp site) are indicated by pictograms or dashed lines. (**b**) Non-metric multidimensional scaling (NMDS) of bacterial communities (n = 47) with the environmental fit (p < 0.01) of porewater properties (grey arrows) based on a weighted generalized UniFrac analysis using the vegan package incorporated into ampvis2^51,57^. Depths are indicated in cm or w (water column) and triangles or circles indicate the sediment core. (**c**) Sampling depths of the sediment cores (Rust Neusiedl RN-K01 and RN-K02) for bacterial community analysis. Each bacterial phylum depicted here comprises more than 1% relative abundance of the bacterial community in at least one sample. All other amplicon sequence variants (ASVs) are summarized as rare taxa and those with a taxonomic match below 95% sequence identity were summarized as “Unclassified”. The phylum *Proteobacteria* is shown at class level (*Alpha-, Gamma-, Deltaproteobacteria*). Names in brackets indicate revised phylum classifications according to Parks *et al*.^28^. The phylogenetic diversity (Faith’s PD) was calculated based on the rarefied community (5,873 reads per sample) and a midpoint-rooted phylogenetic tree. Indicators for microbial activity in the porewater chemistry were selected and depicted as profiles of up to 25 cm depth.

Soft sediment push-cores were taken in the bay of Rust in August 2017 (Fig. 1a). Two 30–40 cm cores were used for bacterial community analysis and one for porewater extraction and analysis. The water (core supernatant) was filtered through a 3.0 and 0.2 µm filter sandwich. All samples for bacterial community analysis were stored in RNAprotect Bacteria Reagent (Qiagen, Hilden, Germany) for transport. The reagent was removed by centrifugation from the samples prior to storage at −80 °C. Metagenomic DNA was extracted from 0.25 g of sediment or one third of a filter. Subsequently, the V3-V4 region of bacterial 16S rRNA genes were amplified using primers described by Klindworth *et al*.^10^. After purification with magnetic beads, the amplicons were sequenced, yielding a total of 6,044,032 raw paired-end reads. Bioinformatic processing of the data included quality-filtering and base pair correction of overlapping regions (fastp), read-merging (PEAR), primer clipping (cutadapt), size-selection, dereplication, denoising and chimera removal (VSEARCH). After taxonomic assignment 2,263,812 high-quality 16S rRNA gene sequences remained in the dataset^11^. Amplicon sequence variants^12^ (ASVs) with 100% sequence identity were screened with BLASTn against the SILVA SSU 132 NR database for taxonomic assignment. The ASV abundance table^13^ was used for visualisation of the bacterial community. Total alkalinity (TA) was determined by titration. Major cations and anions were measured by ion-chromatography and ICP-MS was used to determine trace element content. Nutrient concentrations and total sulphide were assessed photometrically^14^. Porewater chemistry and bacterial community composition were analysed in intervals of 5 cm^15^ (Fig. 1c).

**Fig. 2:**
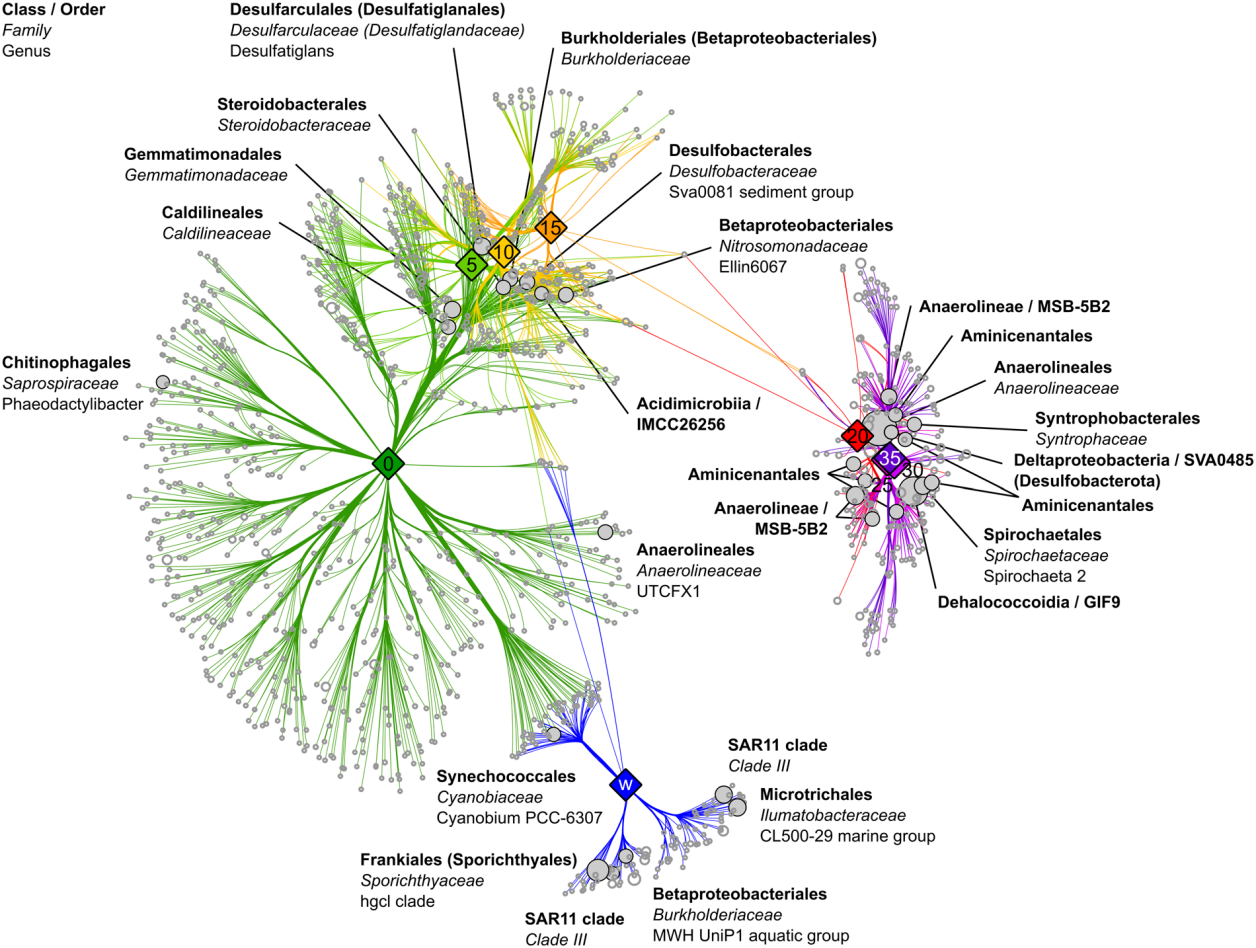
Bacterial genera associated with the different depths of the sediment cores and water column. The association network was calculated with the indicspecies^54^ package in R and visualised in Cytoscape with an edge-weighted spring embedded layout. Branch lengths indicate the phi correlation coefficient. Each light grey circle indicates a bacterial genus associated (p < 0.001) with the depth it is connected to. The 30 most abundant genera are indicated by filled circles and named up to the point where the classification turns to uncultured. Revised names according to Parks *et al*.^28^ are indicated in brackets. Average relative abundance of each genus among all samples is indicated by the circle size. Each sampling depth is indicated by a filled diamond shape containing the depth in cm or w (water column).

The bacterial community composition and diversity as well as the porewater chemistry of the sediment are distinct from the water column and change gradually with depth (Fig. 1b). The water column has a lower phylogenetic diversity than the top sediment layers (Fig. 1c) and is dominated by aquatic *Actinobacteria* (hgcl clade)^16–20^ and freshwater *Alphaproteobacteria* (SAR11 clade III)^16,21–25^ with relative abundances of more than 40% and up to 20% (Figs 1c and 2). The uppermost sediment layer is the most diverse and harbours the largest number of associated genera (Fig. 2). It shares community members of water and sediment, such as *Synechococcus* or the algae-associated *Phaeodactylibacter*^26,27^. The phylogenetic diversity (Fig. 1c) and associated genera (Fig. 2) in the sediment decrease gradually with depth until approximately 20 cm. Members of the *Proteobacteria* and *Chloroflexota*^28^ are dominant in the sediment community, which shifts from 15–35% *Gammaproteobacteria* in the top 15 cm to approximately 40% *Chloroflexota* below 15 cm. Notably, the upper sediment layers harbour sulphate-reducing bacteria, such as *Desulfobacteraceae* and *Desulfarculaceae*^29–32^ (Fig. 2). The decline in sulphate, increase in total sulphide (ΣH_2_S) and low redox potential also indicate sulphate reduction (Fig. 1c). Below 15 cm the bacterial community is associated with *Anaerolineae*, *Aminicenantales* and *Dehalococcoidia* (Fig. 2). Members of these taxa are known fermenters, organohalide respirators and hydrocarbon degraders^33–35^. Increasing degradative processes are indicated by the increase in ammonium and total alkalinity (Fig. 1c).

The bacterial community of Lake Neusiedl has mainly been studied with regard to potential pathogens^6,9^. Here, *Enterobacteriaceae*, more specifically *Escherichia/Shigella*, but not *Vibrionaceae* were detected with a relative abundance of up to 10% at almost all depths in the sediment, but not in the water column. While they indicate an anthropogenic impact on the sediment, the bacteria detected are based on DNA amplification and may not be metabolically active. This data may contribute to studies identifying the sampling site as hotspot for faecal pollution^6,7^ (Fig. 1a). Further, this survey forms a basis for studies targeting biogeochemical cycling in alkaline lakes.

## Methods

### Sediment sampling at Lake Neusiedl, Austria

Three soft sediment push-cores (RN-K01/K02/K03) covering 30 to 40 cm depth were sampled in close lateral distance to each other at the bay of Rust (16°42′33.635″E, 47°48′12.929″N) at Lake Neusiedl, Austria in August of 2017. PVC coring tubes (Uwitec, Mondsee, Austria) of 60 cm length and 63 mm diameter (RN-K01/K03) or 100 cm and 50 mm diameter (RN-K02) were manually pushed into the sediment at the sampling site. A rubber plug was applied to the top of the coring tube to create a partial vacuum, which allowed retrieval of the sediment. After allowing the sediment to settle on cores RN-K01 and K02, 600 ml core supernatant (water column) was filtered through a 3.0 µm polycarbonate (Merck, Darmstadt, Germany) and 0.2 µm polyethersulfone (Sartorius, Göttingen, Germany) filter sandwich. Subsequently, filters were immediately stored in RNAprotect Bacteria Reagent (Qiagen, Hilden, Germany). Sampling of the sediment for community analysis occurred under exclusion of the outer 1 cm of sediment, which is in contact with the walls of the coring tubes. RN-K01/K02 were sampled in triplicate at every 5 cm of depth. RN-K02 was sampled at a higher resolution (every 2.5) as the sediment showed finer lamination. Every triplicate was immediately mixed with RNAprotect Bacteria Reagent (Qiagen, Hilden, Germany) and kept at ambient temperature in a cool box with freezer elements for transport. Before storage, samples were centrifuged at 3,220 × *g* for 15 min and the clear supernatant containing the RNAprotect Bacteria Reagent discarded. Samples were stored at −80 °C. Core RN-K03 and the core supernatant were stored in the cool and dark until analytical chemical analysis.

### DNA extraction and amplification of bacterial 16S rRNA genes

DNA was extracted from 0.25 mg of sediment from each sample of RN-K01/K02 using the MoBio Power Soil Kit (MoBio, CA, USA) with minor modifications. For this purpose, sediments were thawed on ice and homogenized to disrupt any layering caused by the previous centrifugation step. Subsequently, 0.25 mg were transferred into bead-beating tubes supplied by the manufacturer. DNA from the water column (core supernatant) was extracted by cutting one third of the frozen filter sandwiches into small pieces in the bead-beating tubes. After the addition of SDS-containing Solution C1, cells were mechanically disrupted with a FastPrep (MP Biomedicals, Eschwege, Germany) at 6.5 m/s for 20 s. After disruption, the DNA was extracted according to manufacturer’s instructions. Subsequently, DNA was eluted twice in 50 µl of prewarmed DEPC-treated water^36^. Bacterial 16 S rRNA genes were amplified by PCR with forward and reverse primers published by Klindworth *et al*.^10^ and added adapters for MiSeq sequencing (underlined) (D-Bact-0341-b-S-17, TCGTCGGCAGCGTCAGATGTGTATAAGAGACAG CCTACGGGNGGCWGCAG; S-D-Bact-0785-a-A-21, GTCTCGTGGGCTCGGAGATGTGTATAAGA GACAGGACTACHVGGGTATCTAATCC). PCR reactions were performed in a total volume of 50 µl containing 10 µl of five-fold GC Buffer (Thermo Scientific, Waltham, MA, USA), 5% DMSO, 0.2 mM of forward and reverse primer, 200 µM dNTPs, 0.2 mM MgCl_2_, 1 U Phusion High-Fidelity DNA polymerase (Thermo Scientific, Waltham, MA, USA) and 20–25 ng template DNA. The PCR mixture was denatured for 1 min at 98 °C and then subjected to 25 cycles at 98 °C for 45 s, 45 s at 60 °C, and 30 s at 72 °C, followed by a final extension at 72 °C for 5 min. Negative controls were prepared without template and positive controls with genomic *E. coli* DH5α DNA as template. PCR reactions for each sample were performed in triplicate. PCR triplicates were pooled in equal amounts in order to minimize amplification bias, concentrated and purified with MagSi-NGS^Prep^ magnetic beads as recommended by the manufacturer (Steinbrenner, Wiesenbach, Germany). After the final washing step, the beads were air-dried and DNA eluted in 30 µl of elution buffer EB (Qiagen, Hilden, Germany). Purified PCR products were quantified and sequenced as described by Schneider *et al*.^37^ using a MiSeq instrument and v3 chemistry (Illumina, San Diego, CA, USA).

### Bioinformatic processing of 16S rRNA gene amplicons

Paired-end sequencing data from the Illumina MiSeq were quality-filtered with fastp^38^ (version 0.19.4) using default settings with the addition of an increased per base phred score of 20, base pair corrections by overlap (-c), as well as 5′- and 3′-end read trimming with a sliding window of 4, a mean quality of 20 and minimum sequence size of 50 bp. After quality control, the paired-end reads were merged using PEAR^39^ (version 0.9.11) and primers clipped using cutadapt^40^ (version 1.18) with default settings. Sequences were then processed using VSEARCH^41^ (v2.9.1). This included sorting and size-filtering of the paired reads to ≥300 bp (--sortbylength --minseqlength 300), dereplication (--derep_fulllength). Dereplicated amplicon sequence variants (ASVs) were denoised with UNOISE3 using default settings (--cluster_unoise – minsize 8) and chimeras were removed (--uchime3_denovo). An additional reference-based chimera removal was performed (--uchime_ref) against the SILVA SSU NR database (version 132). Raw reads were mapped to ASVs (--usearch_global–id 0.97). The taxonomy was assigned using BLAST 2.7.1+^42^ against the SILVA SSU 132 NR database with an identity of at least 95% to the query sequence resulting in a total of 21,009 ASVs^43^.

### Bacterial community analysis

For data evaluation all samples from the 5 cm intervals were analysed. Additional samples taken due to the finer lamination of one core were not considered in the presented analysis but are available in the dataset^44^. Sequences comprising extrinsic domains, eukaryotes and archaea were removed from the ASV table using grepl, a base R function (version 3.4.4). A phylogenetic tree was generated by aligning all sequences of the filtered dataset with MAFFT^45^ at a maximum of 100 iterations. The tree was calculated using FastTree 2.1.7 (OpenMP)^46^, saved in newick format and midpoint rooted using FigTree^47^ (version 1.4.4).

The dataset was analysed in R^48^ (version 3.4.4) and RStudio^49^ (version 1.1.456). Depth profiles in the form of bar and line charts were generated with ggplot2^50^ (version 3.1.0) using standard R packages. Alphadiversity indices and species richness were calculated with the ampvis2^51^ package (version 2.4.1) and Faith’s phylogenetic diversity with picante^52^ (version 1.7) and the midpoint-rooted tree^15^. For this purpose, 16 samples with a read count below 5,000 were excluded from the diversity analysis (RN17_K1_DNA_Bac_2a, 3a, 5a, 6a, RN17_K2_DNA_Bac_5a-c, 7a-c, 9a-c, 11a-c). All other samples were rarefied in ampvis2 to 5,873 reads. For the visualisation in bar charts, the mean of all replicates from both cores was used to account for the variance at the sampling sites. The non-metric multidimensional scaling (NMDS) matrix was calculated using the ASV table and phylogenetic tree in a weighted generalized UniFrac analysis using the ampvis2 package (version 2.4.1) including the package GUniFrac^53^ (version 1.1). Environmental fit of the metadata were also calculated and plotted onto the NMDS if p < 0.01. An association network of the bacterial community was calculated using the indicspecies^54^ package (version 1.7.6) with the multipatt function and the r.g species-site group association function for calculation of the association strength. The significance cut-off for the phi coefficient was set to p < 0.001. The network was visualised in Cytoscape (version 3.5.1) with an edge-weighted spring-embedded layout using weight as the force and average abundance as the circle size.

### Water column and porewater analysis

For hydrochemical analysis, capped and tightly sealed sediment cores, including the supernatant water column above, were stored upright in the cool and dark until analytical investigation 5 days after sampling. Core supernatants were collected in 250 ml polyethylene (PE) bottles for anion, nutrient, and total alkalinity determination. For cation analysis, a 50 ml aliquot of the supernatants was filtered through 0.7 μm diameter membrane filters (Merck, Darmstadt, Germany) into a PE-bottle and acidified with 100 µl HNO_3_ (suprapure, Merck, Darmstadt, Germany). Physicochemical parameters of the core supernatants were measured using a WTW Multi 3430 device equipped with a WTW Tetracon 925 conductivity probe, a WTW FDO 925 probe for dissolved O_2_, a Schott Pt 61 redox electrode, and a WTW Sentix 940 electrode for temperature and pH, which was calibrated against standard pH-buffers 7.010 and 10.010 (HI6007 and HI6010, Hanna Instruments, Vöhringen, Germany). Total alkalinity (TA) was determined via titration using a hand-held titration device and 1.6 N H_2_SO_4_ cartridges (Hach, Loveland, CO, USA).

Redox potential (Eh) and pH gradients were measured through boreholes directly in the sediment core using a portable WTW 340i pH meter equipped with an Inlab Solids Pro pH-electrode (Mettler Toledo, Gießen, Germany) and a Pt 5900A redox electrode (SI Analytics, Mainz, Germany). Porewater was extracted from core RN-K03 using 5 cm CSS Rhizon samplers (Rhizosphere, Wageningen, Netherlands). Immediately after extraction, aliquots were fixed with Zn-acetate for determination of total sulphide or acidified with suprapure HNO_3_ for analysis of main cations and trace elements. Porewater alkalinity was immediately determined by titration with cartridges (Hach, Loveland, CO, USA) containing self-prepared 0.01 n HCl as titrant. An aliquot for determination of nutrients and anions was stored in the cool and dark until subsequent analysis. Total sulphide (ΣH_2_S) and nutrient concentrations (NH_4_, NO_2_, PO_4_, SiO_2_) were measured by photometric methods according to Grasshoff *et al*.^14^, using an SI Analytics Uviline 9400 spectrophotometer within a few days after extraction.

Major cation (Ca, Mg, Na, K and Li) and anion (Cl, F, Br and SO_4_) concentrations of all water samples (porewaters, water column) were analysed by ion chromatography with non-suppressed and suppressed conductivity detection, respectively (Metrohm 820 IC/Metrosep C3-250 analytical column, Metrohm 883 Basic IC/Metrohm ASupp5-250 analytical column). ICP-MS (ICAP-Q, Thermo Fisher, Waltham, MA, USA) was used to determine Sr, Ba, Fe, Mn, Rb and B, as well as control for the cation determination by ion chromatography. Total dissolved salts (TDS) were calculated as the sum of all measured cations and anions. The chemical analysis was completed within two weeks after extraction with the analytical accuracy of all methods exceeding 1.5%^15^.

All measured values were processed by the PHREEQC software package, version 3^55^, using the phreeqc.dat and wateqf4.dat databases in order to calculate ion activities and *p*CO_2_ (partial pressure of CO_2_) of the water samples and mineral saturation states. The saturation indices of all mineral phases are given as log (IAP/K_SO_) where IAP denotes the ion activity product and K_SO_ is the solubility product of the corresponding mineral (solid phase).

## Data Records

The 16S rRNA gene paired-end raw reads were deposited to the National Center for Biotechnology Information Sequence Read Archive (SRA) and can be found under the accession number PRJNA507590 (Bio Project 507590/SRP171602). This BioProject contains 63 samples and 126 zipped FASTQ files, which were processed using the CASAVA software (Illumina, San Diego, CA, USA)^44^. The processing included demultiplexing and adapter removal from the sequences. The following files have been deposited at figshare: a FASTA file with the assigned ASV sequences after bioinformatic processing^56^; the ASV count table with taxonomic assignments^13^, the read statistics before, during and after bioinformatic processing^11^; the metadata, porewater chemical data and alphadiversity metrics of each sample^15^. The individual files may also be accessed through a figshare collection^43^.

## Technical Validation

For microbial community analysis the layers (2.5–5 cm) of both soft sediment push-cores were sampled in three technical replicates to allow for the microbial heterogeneity at each depth. The PCR reactions were run in three technical replicates per sample and PCR products were pooled equimolar. Negative controls without DNA template and positive controls with genomic *E. coli* DH5α DNA as template were also performed. Correct amplicon size was determined on a 0.8% agarose gel. PCR triplicates per sample were pooled in equimolar amounts for amplicon sequencing to minimize possible PCR bias. Physiochemical data were measured with calibrated probes and ions and nutrients were measured against IC and nutrient standards from Merck (Darmstadt, Germany) and Honeywell Fluka (Charlotte, NC, USA). The analytical accuracy of all methods exceeded 1.5%.

## ISA-Tab metadata file

Download metadata file
